# PI3Kδ as a Novel Therapeutic Target for Aggressive Prostate Cancer

**DOI:** 10.3390/cancers17101610

**Published:** 2025-05-09

**Authors:** Bi-Dar Wang, Alyssa Lucero, Siyoung Ha, Reyhaneh Yarmohammadi

**Affiliations:** 1 Department of Pharmaceutical Sciences, School of Pharmacy and Health Professions, University of Maryland Eastern Shore, Princess Anne, MD 21853, USA; 2 Hormone Related Cancers Program, University of Maryland Greenebaum Comprehensive Cancer Center, Baltimore, MD 21201, USA

**Keywords:** phosphoinositide 3-kinases delta (PI3Kδ), PI3K/AKT/mTOR signaling, PI3Kδ and pan-PI3K inhibitors, combination therapies, splice isoforms, tumor immune microenvironment, immune checkpoint blockers, androgen receptor inhibitors

## Abstract

Phosphoinositide 3-kinases (PI3Ks) signaling represents an important pathway regulating cell proliferation, survival, invasion, migration, and metabolism. Notably, PI3K/AKT/mTOR signaling is frequently dysregulated in the majority of malignancies. Among the class IA PI3Ks (PI3Kα/β/δ), emerging evidence has implicated that PI3Kδ is not only overexpressed in leukocytes but also in solid tumors, including prostate cancer. The critical role of PI3Kδ in tumorigenesis and in the creation of a suppressive tumor microenvironment, along with the recent finding of PI3Kδ splice isoforms in promoting tumor aggressiveness and resistance, further demonstrates the potential of developing novel PI3Kδ-targeted cancer therapies. In this review, we comprehensively describe the functional mechanisms underlying the PI3Kδ-driven tumor progression and immune regulation in prostate cancer diseases. Furthermore, the recent preclinical and clinical studies on the development of PI3Kδ-/PI3K-targeted inhibitors as single agents and in combination therapies (with chemotherapy, radiation, hormone therapy, or immunotherapy) are summarized. Finally, we discuss the potential novel therapies for improving the treatment efficacies, as well as the current limitations and challenges of PI3Kδ-based therapies for prostate cancer.

## Simple Summary

Prostate cancer is the most frequently diagnosed cancer and the second leading cause of cancer deaths among men in the United States. PI3K/AKT/mTOR signaling is frequently upregulated in prostate cancer. Therefore, targeting PI3K or PI3K/AKT/mTOR signaling pathway is considered a promising therapeutic option for treating prostate cancers. In this review article, we comprehensively discuss the oncogenic roles of PI3Kδ (one of the class IA PI3Ks) in the development and progression of prostate cancer. We further summarize the recent progress and future direction on the development of the PI3Kδ-based precision biomarkers and therapeutics for prostate cancer diseases in preclinical models and clinical trials.

## 1. Introduction

Phosphoinositide 3-kinases (PI3Ks) are a family of lipid kinases that play pivotal roles in various cellular processes, including growth, proliferation, and survival [1]. Among the class I PI3K isoforms, PI3Kδ has emerged as a critical player in cancer biology, particularly in the context of immune regulation and tumor progression [2,3,4]. It is well documented that PI3Kδ is highly expressed in leukocytes [5]. Recent studies have further shown that PI3Kδ is also upregulated in solid tumors, correlating with aggressive disease phenotypes and poor patient outcomes [4]. Notably, elevated levels of PI3Kδ expression have been found in prostate, breast, lung, colon and liver cancers, neuroblastoma, and glioblastoma [6,7,8,9,10,11]. Additionally, PI3Kδ expression is upregulated during the progression of breast and liver cancers. Its dysregulation has been implicated in various cancer types, making it a promising target for therapeutic intervention [12]. In hormone receptor-positive breast cancer, the hyperactivation of PI3K (including PI3Kδ) can lead to bypassing the need for estrogen signaling by activating AKT signaling, promoting the estrogen-independent growth and enhancing resistance to hormone (anti-estrogen) therapies [13,14]. Moreover, in non-small cell lung cancer, the activation of PI3Kδ is associated with immune evasion mechanisms, allowing tumors to escape attacks from the immune system [15]. The upregulation of PI3Kδ in colorectal cancer enhances tumor cell metabolism, promotes cell division, and regulates metabolic pathways that enable cancer cells to utilize energy efficiently, impacting tumor growth rates [8,9]. In hepatocellular carcinoma, the chronic inflammation, liver damage, insulin resistance, and metabolic syndrome lead to increased expression of PI3Kδ via NF-κB signaling, promoting cell survival, proliferation, and tumor progression [8]. In prostate cancer (PCa), one of the most prevalent malignancies among men, the upregulation of PI3Kδ has also been documented [4]. Elevated expression levels of PI3Kδ in PCa tissues have been linked to enhanced tumor aggressiveness and resistance to conventional therapies [16]. In addition, the PI3Kδ-mediated pathway is known to interact with multiple downstream signaling cascades, including the AKT/mTOR pathway [17], a crucial signaling pathway for cell survival, growth, proliferation, and cell cycle progression. Furthermore, PI3Kδ is involved in modulating the tumor microenvironment, particularly in the context of immune evasion, further highlighting its significance in cancer progression. This review aims to deliver current knowledge regarding the functional roles of PI3Kδ in PCa, focusing on the following key areas: (1) the specific signaling pathways involving PI3Kδ that contribute to cancer progression; (2) the functional roles of PI3Kδ in PCa; (3) the implications of PI3Kδ splice isoforms in advanced PCa; (4) the interaction between PI3Kδ and the immune microenvironment; and (5) the development of therapeutic strategies through targeting PI3Kδ. Among these topics, recent findings on the oncogenic PI3Kδ splice isoforms in modulating tumor aggressiveness [18] and treatment resistance [19] may also pave new avenues for the development of precision biomarkers and novel therapeutic strategies. Moreover, further exploring the functional roles of PI3Kδ in the immune microenvironment is particularly critical for developing more efficient immunotherapies for PCa, which is known as ‘immune-cold’ tumor [20]. Understanding these complex interactions will further facilitate the development of combination therapies that enhance treatment efficacies against PCa. Finally, we will explore the potential of PI3Kδ inhibitors in combination with chemotherapy, radiation, and immunotherapeutic agents, highlighting the opportunities and challenges associated with the PI3Kδ-based therapies. In summary, this review provides a comprehensive overview focusing on the multifaceted roles of PI3Kδ in PCa, aiming to improve treatment outcomes for patients suffering from this disease.

## 2. PI3Kδ and PI3K Signaling

**Figure 1:**
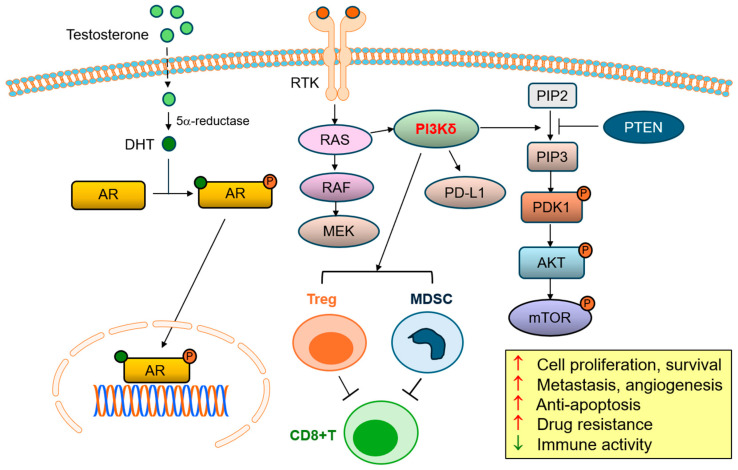
PI3Kδ-mediated signaling cascade in prostate cancer. Overexpression of PI3Kδ stimulates the hyperactivation of AKT/mTOR signaling, cross-interacts with AR signaling, enhances PD-L1 expression, and increases and activates Tregs and MDSCs. Consequently, the PI3Kδ overexpression leads to promotion of cell proliferation, growth, survival, metastasis, angiogenesis, anti-apoptosis, and drug resistance and further creates a suppressive immune microenvironment in prostate cancer. RTK: receptor tyrosine kinase; AR: androgen receptor; P: phosphorylated; Treg: regulatory T cell; MDSC: myeloid-derived suppressor cell; CD8+ T: CD8-positive T lymphocyte.

The PI3K/AKT/mTOR pathway regulates cell growth, differentiation, motility, and survival in human cells. These are vital functions for the onset and growth of cancer [21]. Significant upregulation of the PI3K/AKT/mTOR signaling cascade has been observed in many types of cancers [22,23,24,25]. Among the three class I isoforms, PI3Kδ is frequently involved in both solid tumors and hematologic malignancies [4]. Phosphoinositide 3-kinases (PI3Ks) are categorized into three types (class I, II, and III) based on their structure and substrate specificities. The class I PI3Ks are known to be activated by receptor tyrosine kinases (RTKs) or G protein-coupled receptors (GPCRs) [4,26]. These reactions involve the conversion of phosphatidylinositol 4,5-bisphosphate (PIP2) to phosphatidylinositol 3,4,5-trisphosphate (PIP3), a process catalyzed by PI3K that leads to recruitment and activation of the downstream signaling proteins like AKT [27]. The outcomes of AKT activation include the promotion of cell survival, cell cycle transition, and glucose metabolism. One notable outcome is its effect on the activation of the mammalian target of the rapamycin (mTOR) pathway, leading to activation of cell growth and survival [28]. Class IA PI3Ks are heterodimers containing a 110 kDa catalytic subunit (p110α, p110β, or p110δ) together with one out of five regulatory subunits (including p85α, p85β, p55γ, p55α, or p50α) [29]. Different regulatory proteins form complexes with each catalytic subunit at the plasma membrane for stabilization purposes and modulating its activity. This pathway facilitates the formation of phosphatidylinositol 3,4,5-trisphosphate (PIP3), which binds to various effector proteins and consequently induces cell survival, growth, and motility [30]. PTEN protein acts as a tumor suppressor enzyme that can block this pathway through dephosphorylating and converting PIP3 back to PIP2, thereby inhibiting the PI3K-mediated signal transduction. Loss of PTEN in cancers leads to continuous activation of the class I PI3K pathway. This may be due to the mutations or deletions of *PTEN*, resulting in a decrease or loss of PTEN expression, disruption of protein interactions, or phosphorylation [31,32]. Gain-of-function mutations on the *PIK3CA* gene, which encodes PI3Kα, are found in a broad range of cancers. However, similar mutations in the genes encoding PI3Kβ and PI3Kδ have not been found. Overall, the PI3K/AKT/mTOR pathway represents a critical signaling cascade frequently dysregulated in cancers, making this signaling a promising target for therapeutic intervention [24]. For instance, the inhibitors targeting PI3Kδ have been shown to have the potential to not only constrain tumor growth but also reverse the immune suppression in the tumor immune microenvironment (TIME) [33]. In fact, specific inhibition of PI3Kδ has been demonstrated to decrease immunosuppressive cell populations such as regulatory T cells (Tregs) and myeloid-derived suppressor cells (MDSCs), thus increasing cytotoxic T cell activity and leading to enhanced anti-tumor response [34]. The PI3Kδ-mediated signaling axis is summarized in Figure 1.

Because of the multiple roles played by PI3Kδ in tumor progression and immune regulation, selective inhibitors targeting PI3Kδ have been developed as potential therapeutic agents. These inhibitors hold promise in preclinical studies and are currently under clinical investigations as single agents or in combination with other therapies to enhance their efficacies while retaining specificity towards tumor cells rather than normal ones. The development of the PI3Kδ-based therapies is expected to improve treatment outcomes in PCa and other malignancies, including those involving the immune microenvironment.

## 3. The Functional Roles of PI3Kδ in Prostate Cancer

### 3.1. Specific Role and Signaling Pathways of PI3Kδ in Prostate Cancer

PI3Kδ, although predominantly expressed in immune cells like lymphocytes, has been found to play a critical role in PCa [1,35]. The activation of PI3Kδ in these cells converts PIP2 to PIP3, a key secondary messenger in the signaling pathway [1,36]. PIP3 facilitates the recruitment and activation of AKT through PDK1 (3-phosphoinositide-dependent protein kinase-1) [37]. In PCa, PI3Kδ is integral to other PI3K subunits and is involved in the activation of the AKT/mTOR signaling pathway, which is crucial for tumor cell proliferation, survival, and metabolism. The dysfunction of this pathway is often a result of genetic mutations or overexpression of PI3Kδ, leading to enhanced oncogenic activity [1]. Specifically, mutations in *PIK3CD* and overexpression of PI3Kδ induce the activation of AKT [38], a serine/threonine kinase, subsequently promoting cell growth and survival through the activation of mTORC1 and mTORC2 complexes [39]. Note that mTORC1 primarily regulates protein synthesis and cell growth, whereas mTORC2 is involved in cell survival and cytoskeletal organization [40]. Moreover, the activation of mTORC1 and mTORC2 subsequently leads to the phosphorylation of the downstream targets such as GSK-3β and FOXO transcription factors, promoting cell growth, metabolism, and survival [41,42,43]. In PCa, the overexpression of PI3Kδ results in continuous cell cycle progression and resistance to cell death [44]. Notably, PI3Kδ also plays a functional role in the tumor microenvironment by modulating immune responses and inflammatory processes [45,46]. Additionally, PI3Kδ interacts with the RAS/MAPK pathway, where it activates ERK1/2, promoting cell proliferation and differentiation. PI3Kδ is also involved in the regulation of the NF-κB pathway, a critical signaling pathway for inflammatory responses and cell survival. By modulating NF-κB signaling, PI3Kδ can affect the expression of genes involved in immune responses and apoptosis resistance [17,47]. Together, these integrated actions of PI3Kδ with other pathways contribute significantly to the regulation of the tumor microenvironment. This regulation enhances the progression and metastatic capabilities of cancer by promoting angiogenesis, immune evasion, and stromal support [20,48]. Therefore, understanding these molecular mechanisms is essential for developing novel targeted therapies and immunotherapies against the PCa diseases.

Mutations in the *PTEN* gene (which encodes PTEN, a negative regulator of the PI3K pathway) result in an uncontrolled activation of PI3Kδ [49]. The loss of PTEN function is a common pathologic event in PCa, which significantly contributes to the tumor progression. The hyperactivation of the PI3K pathway, due to PTEN loss, leads to increased cellular proliferation, survival, and resistance to apoptosis in PCa [50]. In addition, PI3Kδ expression levels are inversely correlated with PTEN activities in PCa and other solid tumors. High expression of PI3Kδ is associated with low PTEN activity [10]. DU-145 is a PCa cell line carrying wild-type *PTEN* and overexpressing PI3Kδ. Inhibition of PI3Kδ causes an enhancement of PTEN activity, leading to a reduced phosphorylation of AKT and decreased cell growth in DU-145 cells. Conversely, ectopic expression of *PIK3CD* in PCa cells leads to reduced PTEN activity and increased AKT phosphorylation [10]. MYC is a key oncogenic driver in PCa, and activation of MYC signaling promotes cancer growth and invasion. Previous studies have further revealed that MYC amplification occurs in 10–30% of the localized PCa and more than 50% of the advanced/metastatic PCa cases [51,52]. Amplification/Overexpression of MYC has also been associated with poor prognosis in PCa [53,54]. Activation of PI3K/AKT signaling leads to upregulation of c-MYC signaling, promoting metastasis and contributing to the reduced overall survival of PCa patients [55,56].

Different environmental cues are considered in the PI3K pathway-mediated cellular growth, survival, and metabolism under normal circumstances [28]. In premalignant or transformed cells, hyperactivation of the PI3K pathway offers an adaptive advantage for enhancing biological fitness against various disturbances including stressors, hence promoting the cell survival [57]. Hyperactivation of the PI3K pathway, due to genetic and/or epigenetic alterations, is frequently observed in PCa patients with poor clinical outcomes [58,59]. Dysregulation of the PI3K pathway frequently occurs during prostate tumorigenesis, stimulating enhanced cell growth, proliferation, survival, metastasis, and angiogenesis [60,61]. In PCa, the frequent mutations and/or amplification of *PI3KCA*, activating mutations in *AKT1*, and/or inactivating mutations or the deletion of *PTEN* result in the deregulation of the PI3K signaling pathway [62,63]. Targeting the PI3K pathway, therefore, represents a promising therapeutic option for suppressing tumor progression, metastasis, and drug resistance in PCa [64,65].

### 3.2. Development of Therapeutics Through Targeting PI3Kδ

PI3Kδ inhibitors have demonstrated significant efficacy in treating chronic lymphocytic leukemia (CLL) [66] and have shown great potential as therapeutic agents for solid tumors, including PCa [4,19]. Selective PI3Kδ inhibitors, such as idelalisib (Zydelig, Gilead Sciences, Foster City, CA, USA), specifically target PI3Kδ and result in suppression of the downstream AKT and mTOR signaling pathways [43]. This inhibition disrupts critical processes such as cell proliferation, survival, and metabolism, making these inhibitors promising therapeutic candidates for cancer treatment. Early-phase clinical trials have shown that PI3Kδ inhibitors can reduce tumor growth while enhancing the sensitivity of cancer cells to other therapeutic agents, suggesting a potential for including PI3Kδ inhibitors in the combination therapies [67,68]. The therapeutic landscape of PI3Kδ inhibitors in combination with various anti-tumor agents has demonstrated promising clinical outcomes across different malignancies. For example, a combination of the BTK inhibitor acalabrutinib with the PI3Kδ inhibitor ACP-319 has shown a superior tumor regression capacity in vivo and significantly prolonged survival when compared with monotherapy [69]. The dual inhibition of BTK and PI3Kδ has been shown to be beneficial for patients with high-risk genetic aberrations due to *TP53* mutations and with del(11q) and complex karyotypes, who are the patients that are more likely to fail the single-agent BTK inhibitor therapy [69,70,71,72]. Furthermore, a phase I study of idelalisib combined with rituximab and/or bendamustine in relapsed or refractory indolent non-Hodgkin leukemias (NHLs) showed encouraging efficacy without new toxicity concerns [73]. However, a phase I combination study using idelalisib, lenalidomide, and rituximab in patients with relapsed indolent lymphoma and a phase II study combining idelalisib and entospletinib in relapsed/refractory CLLs and NHLs were terminated early due to unexpected serious toxicities [74]. In addition, a phase I trial, using a combination therapy of dactolisib and abiraterone acetate or everolimus against solid malignancies, has been reported with poor tolerability at the tested doses, highlighting the clinical challenge of balancing efficacy and safety [75,76]. Furthermore, the PI3Kδ inhibitor YY20394, when combined with an anti-PD-1 monoclonal antibody, exhibited a synergistic tumor-suppressive effect in hormone receptor-positive breast cancer (HR^+^ BC) PDX models by modulating Treg activity and restoring sensitivity to immune checkpoint blockade therapy [77]. This finding has suggested the potential of PI3Kδ inhibition to exert anti-tumor effects primarily through the reconstitution of immune surveillance rather than direct induction of cytotoxicity against tumor cells.

These clinical trials are crucial for determining optimal dosing, minimizing adverse effects, and identifying precision biomarkers that predict response to treatment. Furthermore, research is ongoing to develop next-generation PI3Kδ inhibitors with improved specificity and reduced toxicity [78]. These advancements aim to reduce the common side effects associated with PI3Kδ inhibition, such as immune-related adverse events, by refining the selectivity of the PI3Kδ inhibitors against cancer cells over normal cells. In addition to monotherapy, PI3Kδ inhibitors have been evaluated in combination with other targeted therapies, including chemotherapy, radiotherapy, and immunotherapy [67,79,80] (Table 1 and Table 2). Such combinations could potentially overcome the resistance mechanisms that limit the effectiveness of single-agent treatments. For instance, combining PI3Kδ inhibitors with immune checkpoint blockers (ICBs) may enhance anti-tumor immune responses, providing a synergistic effect against PCa [77,81]. Together, the therapeutic potential of PI3Kδ inhibitors extends beyond tumor suppression, and these inhibitors also play a role in modulating the tumor immune microenvironment for further enhancing the efficacies of ICB-based therapies. By inhibiting PI3Kδ, these drugs could reduce the pro-tumorigenic activities of immune and stromal cells, further inhibiting cancer progression [46,82]. Biomarker-driven approaches will facilitate personalized treatment strategies, ensuring that patients receive the most effective therapy based on their unique molecular profiles. As the research progresses, the identification of PI3Kδ overexpressing patients most likely to benefit from PI3Kδ-targeted therapies is becoming increasingly important [83,84]. A previous study has also indicated that PI3K-targeted monotherapies may not be sufficient for achieving significant tumor regression in PCa. Optimal efficacy is likely to be achieved by co-targeting PI3K and other oncoproteins/signaling (such as androgen receptor signaling) in PCa [85]. Overall, the development of PI3Kδ inhibitors represents a promising frontier in the treatment of PCa, with the potential to significantly improve patient outcomes through targeted intervention. The ongoing clinical trials and research efforts are crucial for translating these therapeutic innovations from the laboratory to the clinic setting.

### 3.3. PI3Kδ-Based Combination Therapies

PI3K pathway inhibitors may be beneficial in the treatment of advanced PCa and castration-resistant prostate cancer (CRPC) patients. Because this class of drugs has failed to demonstrate clinical efficacies as monotherapies, combination strategies are warranted. Combined inhibition of the PI3K signaling inhibitors alongside immunotherapy, chemotherapy, and AR inhibitors may offer synergistic effects and favorable outcomes for patients [93]. For instance, significant tumor regression was observed when combining PI3K and AR pathway inhibitors in preclinical PCa models [93]. These PCa cells overexpress PI3Kδ, leading to an inhibition in PTEN activity and consequently activating AKT phosphorylation and promoting cell growth [94]. Specifically, targeting PI3Kδ restores the PTEN function for inhibiting the AKT dephosphorylation, thereby reducing cell growth. Understanding the complex interplay between PTEN and PI3K/AKT signaling in different types of PCa will warrant more effective PI3Kδ-based therapies [94,95]. Some examples of PI3Kδ-based combination therapies (with AR inhibitors, chemotherapies, and immunotherapies) are summarized below.

#### 3.3.1. Combination of PI3K Inhibitors and Androgen Receptor Inhibitors

The PI3Kδ-mediated pathway (such as AKT/mTOR signaling) has also been shown to influence androgen receptor (AR) signaling. Extensive studies have highlighted that PI3K/AKT/mTOR signaling and AR signaling are closely connected in a reciprocal feedback mechanism. Specifically, the activation of PI3K (including PI3Kδ) results in the phosphorylation and activation of AKT. AKT activation further leads to upregulation in AR signaling through direct phosphorylation of AR by AKT and nuclear exclusion of FOXO1 (an AR suppressor) through AKT-mediated phosphorylation of FOXO1. Additionally, AKT activates AR signaling in a cell-type dependent manner. For example, AKT upregulates AR in the androgen-sensitive PCa (such as LNCaP, C4-2B, and 22Rv1) cells, while it represses AR transactivation in androgen-insensitive PCa (such as DU-145, and PC-3) cells transfected with wild-type *AR* [96]. On the other hand, inhibition of AR would lead to hyperactivation of PI3K/AKT/mTOR signaling. Specifically, AR inhibition causes a decrease in FKBP5 level, leading to AKT activation through suppressing the AKT phosphatase PHLPP [97,98].

This reciprocal feedback mechanism between PI3K/AKT/mTOR and AR signaling pathways has led to the development of dual inhibitory approaches for more effective PCa treatments. For example, a systematic examination of the combination of MDV3100 (an AR antagonist) [99] and BKM120 (a PI3K inhibitor) [100], TKI258 (a pan-RTK inhibitor) [101], and RAD001 (an mTOR inhibitor) [102] on various PCa cell lines (LNCaP, VCaP, 22Rv1, PC-3, and DU-145) revealed significant findings. Strong synergy occurred when AR-positive cells were treated with MDV3100 in combination with any of the PI3K pathway inhibitors, including TKI258, BKM120, and RAD001. The growth curve-based synergy assays and Western blot analyses further indicated that the combination of MDV3100 and BKM120 was particularly effective in triggering/promoting the cell death. Additionally, the dual targeting of the PI3K pathway using the BKM120 (PI3K inhibitor) and TKI258 (RTK inhibitor) combination demonstrates a remarkable sensitivity across all five tested PCa cell lines, regardless of their androgen sensitivity. Note that the blockade effects of the BKM120 and TKI258 combination in PC-3 cells are comparable with those observed in combination therapy using BKM120 and cabazitaxel (a next-generation taxane for treating metastatic CRPC) [103].

#### 3.3.2. Combination of PI3K Inhibitors and Immunotherapeutic Agents

PI3K inhibitors were combined with immunotherapeutic agents in several studies. For example, KTC1101 is a novel pan-PI3K inhibitor characterized to target both tumor cell proliferation and the tumor microenvironment. Studies have shown that KTC1101 significantly enhances the efficacies of anti-PD-1 therapy in various preclinical mouse models [104] and in a panel of cancer cell lines, including PC-3, a metastatic PCa cell line [102]. While immunotherapy alone has been unsuccessful in treating PCa, when combined with a PI3K inhibitor, PCa cells have been shown to be sensitized to immunotherapy. Copanlisib, a selective ATP-competitive inhibitor of PI3Kα and PI3Kδ, has been demonstrated to enhance the effect of anti-PD-1antibodies in a prostate-specific PTEN/p-53 genetically engineered mouse (GEM) model. Inhibition of PI3Kα/δ by copanlisib led to a decrease in lactate production, reducing the histone lactylation in tumor-associated macrophages (TAMs). The reduction in histone lactylation activates the TAMs and increases anticancer phagocytosis. PI3K and AR inhibition in combination with immunotherapy led to enhanced phagocytosis compared with a single agent. The combination treatment was found to lead to a reduction in tumor burden and an increase in expression of MHC-II-expressing TAMs in the GEM model. These results suggest that PI3K activation promotes histone lactylation and inhibition of PI3K in combination with immunotherapy promotes anti-tumor phagocytic activity [103].

#### 3.3.3. Combination of PI3K Inhibitors and Chemotherapeutic Drugs

Combination of PI3K inhibitors with conventional chemotherapies have been demonstrated as an effective treatment regimen for PCa. The first-line treatment for mCRPC after the cancer is found to be ADT-resistant is docetaxel; however, the cancer eventually develops multi-drug resistance. A previous study found that the inhibition of PI3K by LY294002 could reverse the paclitaxel resistance in ovarian cancer cells and mice [105]. This finding was replicated in a PCa study. Targeting the PI3K/AKT/mTOR pathway with LY294002 led to sensitize paclitaxel-resistant DU-145 cells to microtubule-targeting chemotherapeutic drugs, such as paclitaxel, docetaxel, and vinblastine. PI3K/AKT and MAPK pathways were upregulated in paclitaxel-resistant DU-145 cells, and the combination of PI3K inhibitors and chemotherapeutic drugs appeared to be a novel therapeutic strategy for overcoming the drug resistance [106].

## 4. PI3Kδ Splice Variants in Prostate Cancer

### 4.1. PI3Kδ Splice Variants That Promote Tumor Aggressiveness and Drug Resistance

Splice variants arise from the alternative splicing of RNA transcripts, producing different isoforms of proteins from the same gene, potentially contributing to their distinct functions and associated regulatory mechanisms [4,105]. For PI3Kδ, its splice isoforms could theoretically alter the enzyme activities, subcellular localizations, interactions with other proteins, or their responses to the inhibitors, which in turn could impact cancer cell behaviors and treatment responses [16,107]. Wang et al. conducted a genome-wide mapping of alternative splicing patterns between African American (AA) and European American (EA) PCa specimens [18]. Their findings revealed that AA PCa harbored both the long (*PIK3CD-L*, full-length transcript containing exon 20) and short (*PIK3CD-S*, splice variant lacking exon 20) variants of *PIK3CD*, whereas EA PCa predominantly expressed *PIK3CD-L*. The activity of PI3 kinase is mainly determined by the efficiency of the ATP binding to amino acid residues K708, M752, D753, S754, W760, I777, and D911 within the ATP binding pocket of PI3Kδ [108]. The *PIK3CD-S* variant, characterized by skipping of exon 20 from the *PIK3CD* pre-mRNA transcript, encodes a splice isoform named PI3Kδ-S. Compared with the full-length PI3Kδ, the PI3Kδ-S splice isoform lacks a 56 amino acid segment, containing three critical binding residues for idelalisib in the hinge and hydrophobic region of the PI3Kδ catalytic domain. Due to the absence of the three critical amino acid residues (E826, V828, and D911), idelalisib (an ATP-competitive PI3Kδ inhibitor) fails to target the ATP binding pocket of the PI3Kδ-S catalytic domain [4,9]. Consequently, cells overexpressing *PIK3CD-S* exhibit a markedly reduced sensitivity to idelalisib and pan-PI3K inhibitors compared with those expressing *PIK3CD-L*, consequently resulting in a resistance to PI3Kδ and PI3K inhibitors. This finding highlights the critical role of *PIK3CD* splice variants in tumor aggressiveness and drug resistance. Idelalisib is an FDA-approved drug for CLL patients. Despite the superior responses found in the clinical trials, ~35% of CLL patients did not respond to the idelalisib therapy, based on the real-world data [109]. Expression levels of *PIK3CD-S* may be correlated to the observed idelalisib resistance in the non-responding CLLs (based on our unpublished data). Therefore, development of novel diagnostic and therapeutic strategies for *PIK3CD-S* expressing cells may warrant the optimal efficacies of the PI3Kδ-based therapies.

### 4.2. Reversing/Correcting Aberrant PIK3CD Splicing in Aggressive PCa

Alternative splicing is a crucial post-transcriptional mechanism that significantly contributes to the proteomic diversity by generating multiple mRNA isoforms from a single gene [109,110]. Among the various isoforms, the PI3Kδ splice variants have garnered considerable attention due to their implications in oncogenesis, particularly in PCa [4]. Recent studies have further elucidated that alternative splicing is predominantly regulated by serine-/arginine-rich splicing factors (SR proteins) [111,112]. Our recent study has further revealed that upregulation of SRSF2 may mediate the alternative splicing of *PIK3CD* pre-mRNA, leading to the synthesis of the *PIK3CD-S* splice variant in AA PCa, as well as other solid tumors [16,113]. Pharmacological targeting SRPK1/2 using SRPIN340 suppresses the SRSF2-mediated exon 20 skipping, thereby effectively reducing the production of the oncogenic *PIK3CD-S* variant [16,113]. This inhibition reverses aberrant *PIK3CD-S* to *PIK3CD-L,* sensitizing the AA PCa cells to PI3K/PI3Kδ inhibitors, suggesting a synergistic approach to targeting *PIK3CD-S*-expressing PCa cells. By targeting SRPK1/2, SRPIN340 inhibits the synthesis of oncogenic splice variants and enhances the overall response to PI3K pathway inhibitors [110]. This finding highlights the potential of SRPIN340 as a valuable addition to current therapeutic strategies aiming to combat PI3Kδ-driven malignancies. Further research and clinical trials are warranted to fully elucidate the long-term efficacy and safety profile of SRPIN340 in combination with PI3K/PI3Kδ inhibitors.

Oligonucleotide technologies are also well considered as promising strategies for targeting aberrant splice variations. Antisense oligonucleotides (ASOs) are short, synthetic strands of nucleic acids designed to bind to specific RNA molecules. Specifically, ASOs can modify splicing patterns, leading to the production of desired splice protein isoforms or preventing the expression of undesired splice isoforms [114]. Development of novel ASOs for inhibiting the generation of *PIK3CD* splice variants may further facilitate novel PI3Kδ-based therapies. Small interfering RNA (siRNA) therapies are another form of RNA-based therapy that can silence specific genes by promoting the degradation of their mRNAs. This can effectively reduce the expression of proteins resulting from splice variants that may contribute to disease. This approach is particularly useful for addressing diseases caused by aberrant splicing [114,115]. SiRNAs specifically targeting *PIK3CD-S* and/or universally targeting *PIK3CD-L* and *PIK3CD-S* variants will theoretically suppress the expression of PI3Kδ isoforms, effectively inhibiting the AA PCa and advanced solid tumors. High-throughput screening (HTS) technology is a method used to quickly assess thousands of compounds for identifying effective compounds with anti-tumor activities. Developing HTS approaches to screen for compounds that specifically target PI3Kδ splice isoforms and/or inhibit tumors expressing PI3Kδ isoforms may warrant the development of novel and effective therapies against AA PCa and advanced PCa in general.

## 5. PI3Kδ and the Immune Microenvironment in Prostate Cancer

### 5.1. The Role of PI3Kδ in Immune Regulation and Prostate Cancer

PI3Kδ is a critical regulator within immune cell signaling pathways, playing a pivotal role in the activation, survival, differentiation, and migration of immune cells [116]. Specifically, PI3Kδ is activated through receptors on T cells and B cells, modulating their functions by either enhancing or suppressing immune responses [117,118]. PI3Kδ activity is tightly regulated by upstream signals such as cytokines and antigen receptors, ensuring that immune responses are precisely controlled [119,120]. Dysregulation of PI3Kδ activity can lead to aberrant immune responses, contributing to autoimmune diseases and malignancies [45,52]. PI3Kδ plays a critical role in regulating immune cell functions, particularly in the suppression of CD8+ T cell activity. Activation of PI3K in tumor cells leads to increased expression of PD-L1, which binds to CD8+ T cells and inhibits their activation through immune checkpoint mechanisms, thereby dampening the immune response and promoting tumor cell survival [20]. Additionally, PI3Kδ promotes the activation and function of Treg cells, which secrete immunosuppressive cytokines such as IL-10, TGF-β, and IL-35, further inhibiting CD8+ T cell function. PI3Kδ is also involved in the activation of myeloid-derived suppressor cells (MDSCs) and M2 macrophages, creating an immunosuppressive environment that supports tumor growth. Additionally, PI3Kδ can lead to the depletion of IL-2, a crucial cytokine for T cell survival and proliferation, thereby inactivating CD8+ T cell function [20]. Therefore, targeting PI3Kδ may offer new opportunities for enhancing anti-tumor immune responses by restoring the activity of CD8+ T cells. The PI3Kδ inhibitors can reactivate T cells by reversing the immunosuppressive effects mediated by Tregs and MDSCs, both of which express PI3Kδ [121,122]. Indeed, these inhibitors have the potential to enhance immune cell activation and reshape the immunosuppressive tumor microenvironment, further enhancing anti-tumor immune responses [121,122]. In the context of PCa, overexpression of PI3Kδ contributes to an immunosuppressive tumor microenvironment that supports tumor cell survival and growth [50,51]. This is particularly evident through the inactivation of T cells and the overexpression of immune checkpoint molecules such as PD-1 and CTLA-4 [123,124]. The tumor-promoting effects of PI3Kδ are mediated by its ability to modulate various cellular processes, including the inhibition of apoptosis and enhancement of cell proliferation [82]. Moreover, PI3Kδ influences the recruitment and activation of Tregs and MDSCs, both of which play crucial roles in suppressing anti-tumor immune responses. These immune cells can produce cytokines and growth factors that further support tumor progression and metastasis [83]. Additionally, as described in Section 3.2, PI3Kδ inhibitors can synergize with immune checkpoint blockers (ICBs, such as PD-1/PD-L1 inhibitors), providing a more robust and sustained anti-tumor response [78,125]. This approach (combination of PI3Kδ inhibitors and ICBs) aims to overcome the immune resistance mechanisms developed from the single-agent therapies [126], enhancing the overall efficacies against tumors, specifically immunotherapy-resistant cancers, such as PCa [126].

PI3Ks are a family of enzymes that play crucial roles in various cellular processes, including growth, proliferation, and survival. Among these, PI3Kδ has emerged as a significant player in immune regulation, particularly within the tumor immune microenvironment (TIME) of PCa [127]. PI3Kδ is predominantly expressed in leukocytes, where it is essential for the proper functioning of several immune cell types, including T cells, B cells, and myeloid lineages [128]. In T cells, PI3Kδ mediates critical signaling pathways that facilitate activation, survival, and differentiation [129]. Specifically, it is involved in the signaling cascades initiated by the T cell receptor (TCR), which are crucial for T cell activation. One of the most significant roles of PI3Kδ in regulating T cell activity is through Tregs [130]. Tregs are vital for maintaining immune homeostasis and preventing autoimmunity [131]. However, in the context of cancer, Tregs can suppress the activity of cytotoxic T lymphocytes (CTLs) infiltrating tumors, allowing cancer cells to evade immune surveillance [132,133]. Increased infiltration of Tregs into the PCa TIME creates an immunosuppressive environment that significantly impairs the effectiveness of immunotherapies, such as ICB-based therapies [126,133,134].

### 5.2. Immune Regulation and Prostate Cancer Therapy by PI3Kδ Inhibitors

Similarly, the TIME of PCa is characterized by a complex interplay between cancer cells and various immune cells, including Tregs and MDSCs [122,135]. PI3Kδ signaling has been shown to promote the accumulation of these immunosuppressive cell types in the TIME [38], thereby fostering an environment conducive to tumor growth and progression. MDSCs, which are known to inhibit T cell activation and promote tumor growth, are particularly influenced by PI3Kδ activity [38,82,136]. The presence of MDSCs in the TIME correlates with poor prognosis in PCa patients, highlighting the detrimental effects of PI3Kδ on anti-tumor immunity [137,138]. This suggests that higher levels of MDSCs may contribute to disease progression and reduced survival rates [122]. Additionally, it emphasizes the harmful effects of the PI3Kδ signaling pathway on anti-tumor immunity. Specifically, PI3Kδ may promote the accumulation and activity of MDSCs [82], which in turn suppresses the immune response against tumor cells [44]. Thus, targeting this pathway could be crucial for improving immune responses and patient outcomes in PCa [81].

Recent studies have demonstrated that inhibiting PI3Kδ can reverse the immunosuppressive effects mediated by Tregs and MDSCs [82,136], enhancing the efficacy of existing therapies. For example, the use of PI3Kδ inhibitors in combination with immunotherapies has shown promise in preclinical models and early-phase clinical trials [139]. These inhibitors can help restore T cell function and promote a more favorable immune response against tumors [20]. In particular, PI3Kδ inhibitors have been shown to decrease Treg populations and reduce their suppressive activity [86], allowing a more robust activation of the immune system against PCa cells [81]. This strategy not only enhances the efficacies of immunotherapies but also has the potential to improve clinical outcomes in PCa patients resistant to conventional therapies.

## 6. PI3Kδ Therapy: Opportunities and Challenges

### 6.1. Advancement of PI3Kδ-Based Therapeutic Strategies in Cancers

PI3Kδ inhibitors are currently being evaluated in multiple clinical trials for their efficacy as treatments for various cancers, including PCa [76,140,141]. Emphasizing the identification of prognostic biomarkers and patient subsets will most likely facilitate the development of more effective PI3Kδ-based therapeutic strategies [4,19]. Clinical trials have been rigorously evaluated for understanding the efficacies, safeties, and optimal doses of the tested PI3Kδ inhibitors. These PI3Kδ inhibitors were also integrated into existing treatment paradigms, such as chemotherapy or radiotherapy, to enhance therapeutic outcomes [142]. Furthermore, the exploration of combining PI3Kδ inhibitors with other immunotherapies marks a pivotal shift towards personalized medicine, aiming to enhance treatment efficacies, eradicate residual disease, and prevent cancer relapse and metastasis [143,144]. Advances in genomic and proteomic technologies are pivotal in defining the patient selection for PI3Kδ-based therapies, with a keen focus on understanding the long-term immunological and health benefits of PI3Kδ inhibition. This comprehensive approach seeks to develop synergistic combination therapies that boost anti-tumor immunity while mitigating adverse effects, ultimately aspiring to increase survival rates and quality of life for PCa patients.

The integration of PI3Kδ inhibitors into other treatment regimens for PCa represents a promising strategy to enhance the treatment efficacies and overcome resistance for the existing therapies. This novel approach aims to enhance treatment efficacy by addressing the underlying mechanisms that contribute to therapeutic failure. Combining PI3Kδ inhibitors with traditional therapies, such as androgen receptor (AR) inhibitors, chemotherapy, radiation, and immunotherapy, has shown potential in preclinical studies [85,86,139,145,146,147]. These combinations may sensitize the PCa cells to treatment through modulating immune responses and reducing tumor cell survival. Clinical trials are currently conducted to evaluate the safety and efficacy profiles of these combinations, with the goal of establishing a new standard of care for patients with advanced or resistant PCa [88,148,149]. Understanding the specific drug resistance mechanisms driven by PI3Kδ would facilitate the development of targeted therapeutic strategies for aggressive PCa. Identifying biomarkers associated with PI3Kδ activity is crucial for predicting how patients will respond to therapies. For instance, overexpression of PI3Kδ (and PI3Kδ splice isoforms) in tumors may indicate a higher risk of treatment resistance to certain treatments, and more personalized approaches should be administrated. Biomarkers can also help stratify patients for clinical trials, ensuring that those most likely to benefit from PI3Kδ inhibition are prioritized. The insights gained from studying PI3Kδ can lead to more personalized treatment regimens for PCa patients. By tailoring therapies based on individual tumor genomic profiles and the PI3Kδ activities [150], clinicians can tailor personalized treatment plans to improve the outcomes [22]. This approach not only holds the potential to enhance efficacy but also to minimize unnecessary side effects and avoid ineffective treatments.

### 6.2. PI3Kδ-Based Therapies in Preclinical Models

In preclinical studies, the pan-PI3K inhibitor dactolisib, a dual ATP-competitive pan-PI3K and mTOR inhibitor, in combination with enzalutamide has been shown to effectively induce tumor regression in the MYC-overexpressing and PTEN-deficient GEM model [87]. AZD8186, a selective competitive ATP inhibitor against PI3Kβ and PI3Kδ, has been shown to effectively suppress tumor growth in the PTEN-deficient cancer cell lines (MDA-MB-436 and MDA-MB-468)-derived xenografts, particularly when combined with paclitaxel [147,151]. AZD8186 has been involved in multiple clinical trials in treating PCa, specifically mCRPC, and other solid tumors. Simultaneous targeting of the PI3K/AKT and AR pathways with the combination of copanlisib and darultamide was found to decrease the tumor burden of SCID mice with LuCaP 35 PDX tumors [88]. Specifically, the dual inhibition led to a decrease in tumor volume and the IHC staining indicated an increased expression of apoptosis markers, cleaved caspase 3, and BBC3. Additionally, BAY1082439 (an ATP-competitive PI3Kα/β/δ inhibitor) was applied to reverse the immunosuppressive microenvironment in PCa by promoting the activation of IFNα and IFNγ. Intermittent treatment of BAY1082439 on castrated mice inoculated with PTEN-null PCa cells was found to promote the expansion of CD8+ T cells and sensitize the PTEN-negative PCa xenografts to the anti-PD-1 immunotherapy (Table 1) [44].

### 6.3. PI3Kδ/PI3K-Based Therapies for Prostate Cancers in Clinical Trials

Preclinical data suggest that overexpression of PI3Kδ may lead to the development and progression of PCa. Clinical studies have been employed to evaluate the anti-tumor efficacies of targeting PI3Kδ in PCa patients.

AZD8186, a selective inhibitor for PI3Kβ/δ, was assessed in several clinical trials for its therapeutic potential in treating solid tumors. Clinical trial NCT04001569 by Seoul National University was a phase Ib/II clinical study to assess the therapeutic efficacy of AZD8186 in combination with paclitaxel. The safety profile of the combination of AZD8186 with paclitaxel indicated that the treatment was well tolerated; however, the trial ended at phase II due to poor clinical efficacy in advanced gastric cancer patients with mutated *PTEN.* However, the patients with *PIK3CB* mutations had greater long-term clinical benefits than their counterparts, indicating that AZD8186-based therapy may have greater clinical efficacy in a small subset of the patient population (i.e., patients overexpressing *PIK3CB*), but not in all cases of PCa [89]. In addition, a phase I clinical trial (NCT01884285) by AstraZeneca assessed the effective dose and preliminary efficacy of AZD8186 alone or in combination with abiraterone acetate or AZD2014. The study is in patients with metastatic CRPC (mCRPC), triple-negative breast cancer, squamous non-small cell lung cancer, and *PTEN*-null or *PIK3CB-*mutated solid tumors. The monotherapy and combination therapies were found to have an acceptable safety profile and showed preliminary evidence of anti-tumor activity in prostate cancer [90]. Similarly, NCI has a clinical trial (NCT03218826) to study the effective dose and safety profile of AZD8186 in combination with docetaxel in *PTEN-* or *PIK3CB-*mutated solid tumors. BKM120 is an orally available pan-PI3k inhibitor that inhibits multiple isoforms of PI3K, including PI3Kδ. NCT01385293, a phase II trial conducted by Duke University, evaluated the therapeutic efficacy of BKM120 in men with mCRPC who had cancer progression following docetaxel treatment. However, the trial was terminated due to futility as the results indicated that BKM120/enzalutamide combination did not have a significant therapeutic effect in men with mCRPC. A factor that may influence the treatment efficacy may be due to a drug–drug interaction effect (as enzalutamide has been shown to induce cytochrome P450), possibly increasing drug metabolism and reducing the treatment efficacies [92]. A similar clinical trial (NCT02035124) by SCRI Development Innovations was proposed to evaluate the efficacy of BKM120 in combination with cabazitaxel in post-docetaxel-treated men with mCRPC. However, that trial was withdrawn due to slow accrual and short of patient enrollment. Besides PI3K inhibitors, dual-target inhibitors have also been developed that target both PI3K and mTOR for treating PCa. LY3023414 is a potent ATP-competitive PI3K/mTOR dual inhibitor. In a clinical trial (NCT02407054) conducted by Eli Lilly and Company, patients with mCRPC progression following abiraterone treatment were recruited. These mCRPC patients were treated with enzalutamide alone or in combination with LY3023414. In this trial, enzalutamide in combination with LY3023414 showed an acceptable safety profile and improved progression-free survival in CRPC patients previously treated with abiraterone [91]. In addition, gedatolisib, another ATP-competitive PI3K/mTOR dual inhibitor, is also under investigation in a phase 1/2 clinical trial (NCT06190899). This study intends to find the effective dose and investigate the preliminary effect of gedatolisib in combination with darolutamide in men with mCRPC who have previously undergone androgen deprivation therapy. Taken together, these clinical trials indicate a growing interest in PI3Kδ-targetd therapies against PCa, particularly among the mCRPC patients who did not respond to the hormone therapies or chemotherapies (Table 2).

## 7. Conclusions

This review has provided a comprehensive overview of PI3Kδ functions in cancer development, progression, and evasion of immune surveillance. As the research progresses, a deeper understanding of the interactions between PI3Kδ and other signaling pathways in PCa will be essential. Investigating potential novel therapies (by compounds directly targeting PI3Kδ isoforms, or ASOs/siRNAs targeting/correcting *PIK3CD* variants) and synergies of PI3Kδ-based therapies with emerging therapies, such as immune checkpoint inhibitors and novel targeted agents, could further improve treatment outcomes. Additionally, exploring the role of the tumor microenvironment and its interaction/regulation with PI3Kδ may facilitate the development of new therapeutic targets and strategies. In summary, the clinical implications of integrating PI3Kδ inhibitors into current treatment regimens for PCa are promising. By enhancing the effectiveness of existing therapies, utilizing biomarkers for personalized treatment, and exploring future research directions, there is great potential for further advancements in the PI3Kδ-based management for the advanced and treatment-resistant PCa, and ideally, all tumors overexpressing PI3Kδ.

## Figures and Tables

**Table 1:** PI3Kδ-based therapies tested in PCa preclinical models.

Drug	MOA ^a^	Target	Cell Line	Treatment	Preclinical Model	Reference
AZD8186	Selective ATP-inhibitor of PI3Kβ and PI3Kδ	PI3Kβ PI3Kδ	Prostate and breast cancer cell lines	AZD1816 was combined with MCL-1 inhibitor AZD5991 to treat the nude mice injected with *PTEN*-deficient breast and prostate cancer cells	Athymic *Foxn1nu* nude mice, xenograft models	[86]
Dactolisib	Dual ATP-competitive inhibitor of pan-PI3K and mTOR	PI3K	Prostate Cancer	Dactolisib was combined with enzalutamide to treat PTEN-null prostate-specific genetically engineered mice.	PB-*MYC* and *Pten*^lox/lox^ PB-*Cre* mice, GEM models	[87]
Copanlisib	Selective ATP-competitive inhibitor of PI3Kα and PI3Kδ	PI3K	Prostate Cancer	Copanlisib was combined with darolutamide to treat LuCaP (PDX) mice	Fox Chase SCID mice, PDX model	[88]
BAY1082439	Selective ATP-competitive inhibitor of PI3Kα/β/δ	PI3Kα PI3Kβ PI3Kδ	Prostate Cancer	BAY1082439 was combined with anti-PD-1 therapy to treat PTEN-null mice	*Pb-Cre*^+^; *Pten^LoxP/LoxP^*; *Cd8*-KO (*Pten*-null; *Cd8*-KO) mice, GEM models	[44]

^a^: Mechanism of action.

**Table 2:** PI3Kδ/PI3K therapies in clinical trials for prostate cancer.

Drug	MOA	Clinical Trial ID	Phase	Treatment	Target	Reference
AZD8186	Selective ATP-inhibitor of PI3Kβ and PI3Kδ	NCT04001569	Phase Ib/II	AZD8186 alone or in combination with paclitaxel were used for treating advanced cancer patients with *PTEN* deficiencies	PI3Kβ PI3Kδ	https://clinicaltrials.gov/study/NCT04001569 (accessed on 24 January 2025) [89]
		NCT01884285	Phase I	AZD8186 alone or in combination with abiraterone acetate or AZD2014 were used to treat mCRPC patients with PTEN deficiencies or *PIK3CB* mutations	PI3Kβ PI3Kδ	https://clinicaltrials.gov/study/NCT01884285 (accessed on 24 January 2025) [90]
		NCT03218826	Phase I	AZD8186 alone or in combination with docetaxel were used to treat the advanced solid tumors carrying *PTEN* or *PIK3CB* mutations	PI3Kβ PI3Kδ	https://clinicaltrials.gov/study/NCT03218826 (accessed on 24 January 2025)
LY3023414	ATP-competitive inhibitor of pan-PI3K and mTOR	NCT02407054	Phase II	LY3023414 combined with docetaxel for treating mCRPC patients resistant to abiraterone treatment	PI3K mTOR	https://clinicaltrials.gov/study/NCT02407054 (accessed on 24 January 2025) [91]
BKM120	ATP-competitive inhibitor of pan-PI3K	NCT01385293	Phase II	BKM120 alone or in combination with enzalutamide to treat in mCRPC previously treated with docetaxel. Note: this trial was terminated due to futility.	PI3K	https://clinicaltrials.gov/study/NCT01385293 (accessed on 24 January 2025) [92]
		NCT02035124	Phase II	BKM120 in combination with cabazitaxel, compared with cabazitaxel alone, in patients with mCRPC. Note: this trial was withdrawn due to poor enrollment.	PI3K	https://clinicaltrials.gov/study/NCT02035124 (accessed on 24 January 2025)
Gedatolisib	ATP-competitive inhibitor of pan-PI3K and mTOR	NCT06190899	Phase I/II	Gedatolisib combined with darolutamide to treat mCRPC resistant to previous treatment using AR inhibitors.	PI3K mTOR	https://clinicaltrials.gov/study/NCT06190899 (accessed on 24 January 2025)

## Data Availability

Not applicable to this study.
